# Bridge of the left anterior descending artery revealed by syncope: a case report

**DOI:** 10.1097/MS9.0000000000000508

**Published:** 2023-04-05

**Authors:** Mohammed Boutaybi, Ikram Tahani, Mohammed EL-Azrak, Nabila Ismaili, Noha EL Ouafi

**Affiliations:** a Department of Cardiology, Mohammed VI University Hospital; b Epidemiological Laboratory of Clinical Research and Public Health, Faculty of Medicine and Pharmacy of Oujda, Mohammed First University, Oujda, Morocco

**Keywords:** atrioventricular block, bridge, congenital coronary anomaly, syncope

## Abstract

The myocardial bridge is a congenital coronary anomaly defined as the presence of a region of myocardium overlying an epicardial coronary artery. This is a 51-year-old patient, diabetic for 4 years on oral hypoglycemic, has had stress angina for 4 years, neglected by the patient. The current history goes back to 2 months by the installation of an episode of syncope occurring with the effort, then of a second episode the day of its admission. Electrocardiogram on admission showed complete atrioventricular block with an heart rate of 32 beats per minute, the patient spontaneously recovered sinus rhythm with a heart rate of 88 beats per minute and a PR interval of 200 ms, coronary angiography was performed showing coronary arteries without stenosis with an intramyocardial bridge of the left anterior descending artery. During exercise and in the presence of a myocardial bridge on the left anterior descending artery, systolic compression leads to a decrease in flow to the septal branches, which is responsible for an alteration of the vascularization of the sub-nodal tissue with paroxysmal conduction disorders leading to syncope. Conduction disorders of ischemic origin are not always associated with atherosclerotic or thromboembolic lesions, but may also be secondary to myocardial bridges.

## Introduction

HIGHLIGHTS

- The myocardial bridge is a congenital coronary anomaly.
- The left anterior descending artery represents the coronary branch most prone to this anomaly.
- The treatment consists of medical, interventional, or surgical management.

The myocardial bridge is a congenital anomaly first described by Reyman in 1737, defined by the presence of an area of myocardium overlying an epicardial coronary artery.

Patients with myocardial bridges are usually asymptomatic, but this congenital anomaly may be associated with acute or chronic coronary syndromes, rhythm or conduction disorders, syncope, or even sudden death.

Treatment consists of medical, interventional, or surgical management.

This case report has been reported in line with the Surgical Case REport (SCARE 2020) criteria^1^.

## Case report

**Figure 1:**
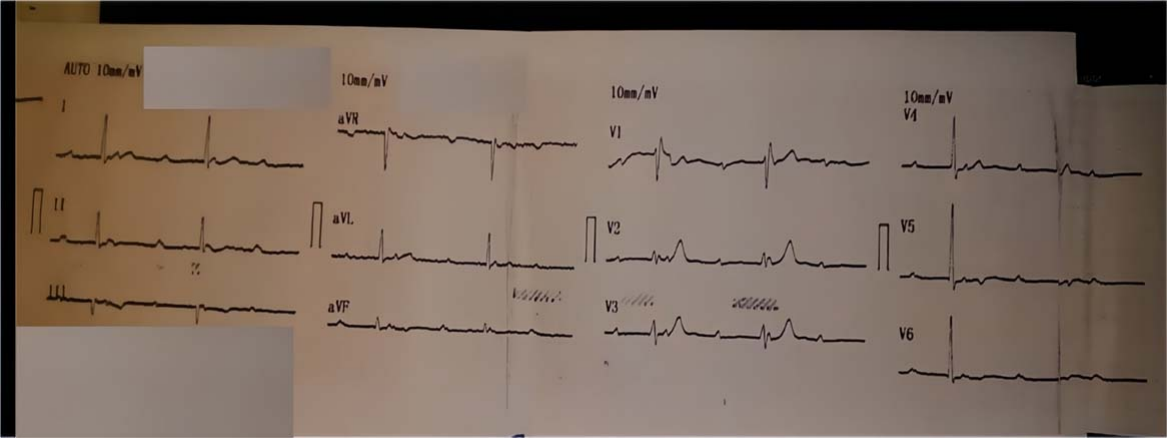
Electrocardiogram showing complete atrioventricular block.

**Figure 2:**
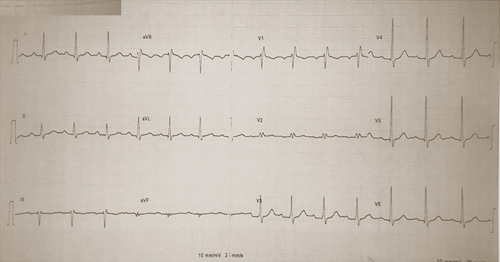
Electrocardiogram showing sinus rhythm with PR at 200 ms.

**Figure 3:**
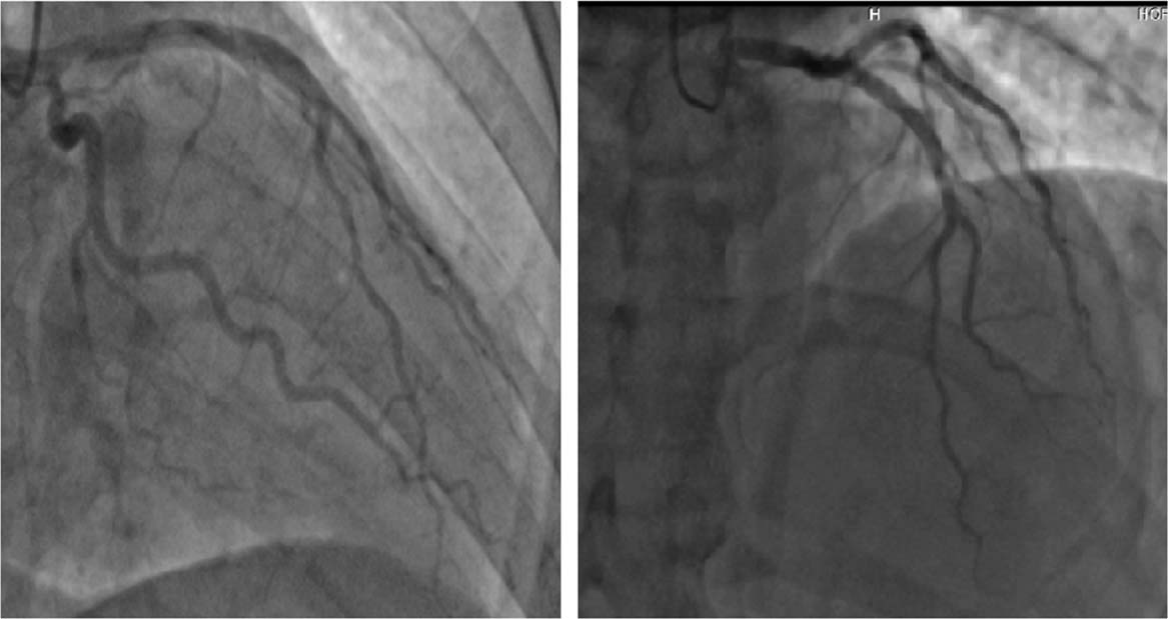
Coronary angiography showing the left anterior descending artery without stenosis.

**Figure 4:**
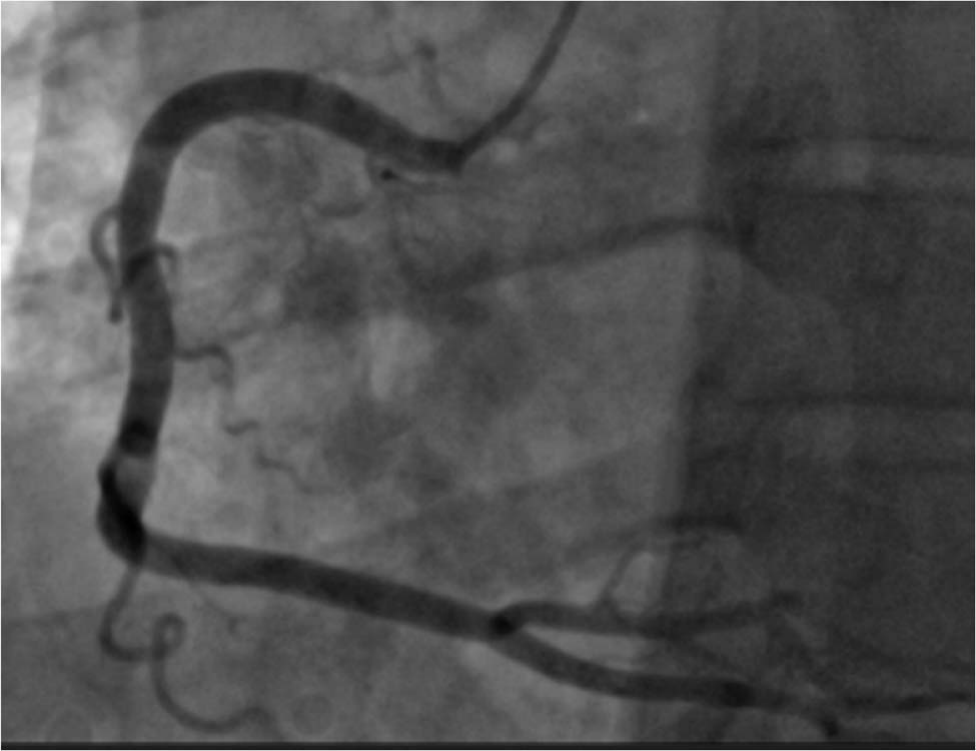
Coronary angiography showing right coronary artery without stenosis.

**Figure 5:**
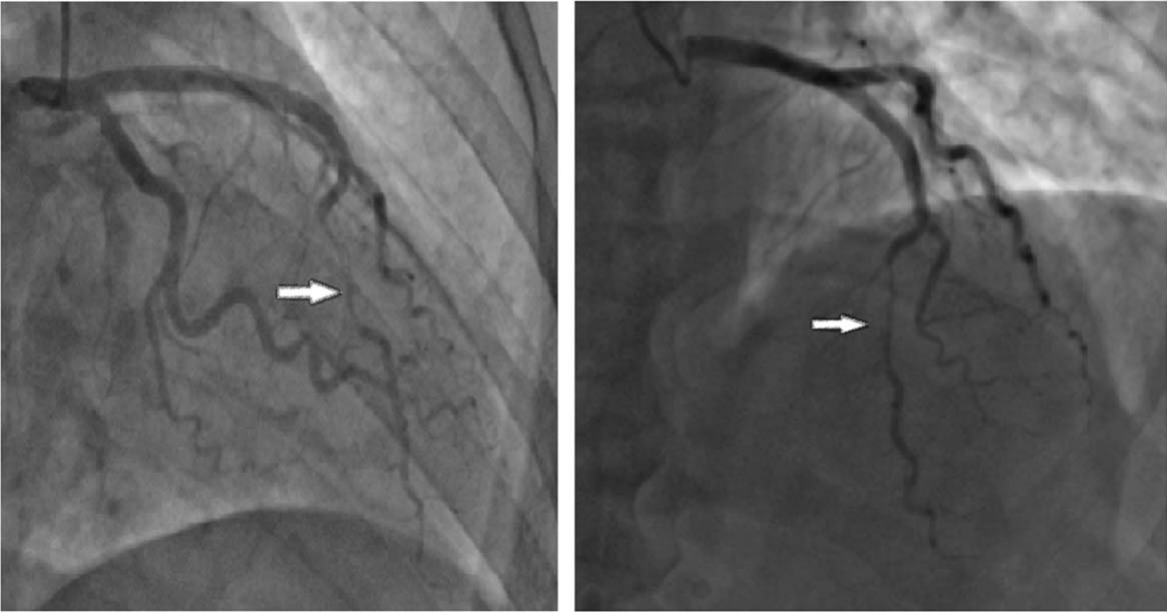
Coronary angiography showing a left anterior descending artery bridge.

A 51 years old man, diabetic for 4 years on oral hypoglycemic, without a medical history, and without a particular family history, has had stress angina for 4 years, neglected by the patient. Current history goes back 2 months by the installation of an episode of syncope occurring with the effort, then a second episode the day of its admission. The clinical examination on admission finds a conscious patient, blood pressure at 130/70 mmHg, bradycardia with a heart rate (HR) at 32 beats per minute, the oxygen saturation was at 96% in room air, and cardiac and pulmonary examinations were normal. A 18-leads resting electrocardiogram shows complete atrioventricular (AV) block with a HR of 32 beats per minute, (Fig. 1). The patient spontaneously recovered sinus rhythm with a HR of 88 beats per minute and a PR interval of 200 ms, without rhythm or conduction disturbances (Fig. 2). Transthoracic echocardiography was performed, showing normal global and segmental left ventricular systolic function (ejection fraction at 69%), no mitro-aortic valvuloplathy, a right ventricle of normal size and systolic function (tricuspid annular plane systolic excursion: 18 mm; S wave: 0,11 m/s) with a dry pericardium. The biological check-up has shown a correct renal function with a creatinine level at 6 mg/l, kalemia at 3.7 mmol/l, a normal thyroid check-up with TSH us at 1.5 mUI /l, a negative troponin, a CRP at 6 mg. Other results are summarized in (Table 1). Coronary angiography was performed showing coronary arteries without stenosis (Figs. 3 and 4) with an intramyocardial bridge of the left anterior descending artery (LAD). (Fig. 5). The patient was put on a calcium channel blocker with a good evolution without recurrence of syncope over a follow-up period of 10 months. Holter electrocardiogram of control was realized objectifying a sinusal rhythm without rhythm or conduction disorder.

**Table 1:** Significant laboratory findings

Examen	Results	Normal values
Albumin (g/l)	43.00	34–54
C-reactive protein (mg/l)	6	6–12
Urea (g/l)	0.22	<0.45
Creatinin (mg/l)	6	(6–12)
Potassium (mmol/l)	3.7	(3–5)
Natremia (mmol/l)	141	(135–140)
Troponin Level (ng/mL)	5	< 26
White blood cells (E/mm3)	7500	(4000–10 000)
Hemoglobin (g/dl)	14	> 13
Hematocrit	51.2	40–52
Platlets	178000	(150 000–400 000)
TSH us (mUI)	1,5	(0,5–4)

## Discussion

The first radiological description of myocardial bridges was given by Portsmann and Iwig in 1960. Before that, Geiringer gave an autopsy description in 1951, and Reyman gave the first anatomic description in 1737^2^.

Its prevalence varies between series. It is less than 5% in angiographic series and 5–86% in autopsy series^3^-^5^. Myocardial bridges have a higher incidence in obstructive hypertrophic cardiomyopathy^4^, and are often multiple and severe. Corban *et al.* 5 reported that 67–98% of myocardial bridges were located in the LAD.

The myocardial bridge is a congenital coronary anomaly defined by the presence of a region of myocardium overlying the epicardial coronary arteries such that the coronary arteries have an intramyocardial pathway^6^. Contraction of the myocardium during systole results in compression of the epicardial arteries, in most cases, this systolic compression is silent^7^ because coronary perfusion is diastolic. In its symptomatic form, it is manifested by acute or chronic coronary syndromes^8^, ventricular or supra-ventricular arrhythmias, conduction disorders suggestive of ischemic mechanisms, syncope, or even sudden death^9^.

The vascularization of the sinus node is ensured by the sinus node artery, which originates from the right coronary artery in 64% of cases, and from the left coronary artery in 36% of cases (most often from the circumflex artery) in patients with left coronary dominance^10^. The vascularization of the AV node is ensured by the AV node artery, a branch of the right coronary artery in 90% of cases, of the left coronary artery in 10% of cases^11^. Ischemic conduction disturbances are mainly seen in acute lower territory myocardial infarction, where the right coronary artery is often the culprit artery, high-grade AV block described in up to 17% of cases^12^, the majority of these cases are transient, while about 9% require a permanent pacemaker due to permanent nodal tissue damage^13^. Coronary angiography in our patient identified a coronary network without significant stenosis, which excluded atherosclerotic or thrombotic causes of the observed conduction disturbances.

Arterial vascularization of the sub-nodal conduction system is provided almost entirely by the septal branches of the LAD^14^. In the presence of a myocardial bridge over the LAD, systolic compression results in decreased flow to the septal branches, which are responsible for impaired vascularization of the subnodal tissue resulting in secondary conduction disorders.

During exercise, tachycardia shortens the diastolic filling time, increases myocardial contractility, which increases the degree of compression and prolongs the obstructive effect of the tunneled segment, resulting in reduced cardiac output on the one hand and impaired vascularization of the sub-nodal tissue on the other. This results in low cerebral blood flow and paroxysmal conduction disorders, leading to syncope on exertion^15^.

The treatment of myocardial bridging consists of medical, interventional, or surgical management. For patients with symptomatic myocardial bridges, beta-blockers are the first-line treatment in the absence of contraindications. They lower the HR and promote an increase in ventricular diastolic time and a decrease in systolic compression^16^. Calcium channel blockers are used when beta-blockers are contraindicated or when vasospasm is suspected. Percutaneous coronary intervention with stenting can be considered for symptomatic improvement, but results in a high rate of restenosis and other complications. Myotomy or coronary artery bypass grafting may be the treatment of choice in patients with symptomatic myocardial bridges resistant to medical therapy^17^.

## Conclusion

Myocardial bridging is a congenital coronary anomaly characterized by an intramyocardial course of an epicardial coronary artery.

The LAD represents the coronary branch most prone to this anomaly.

Usually asymptomatic, but clinical and electrical signals reflecting disturbances of myocardial perfusion, rhythm, or conduction may occur.

Conduction disorders of ischemic origin are not always associated with atherosclerotic or thromboembolic lesions, but may also be secondary to myocardial bridges.

## Ethical approval

NA.

## Consent

Written informed consent was obtained from the patient for publication of this case report and accompanying images. A copy of the written consent is available for review by the Editor-in-Chief of this journal on request

## Sources of funding

No funding was received for this work.

## Author contributions

N.E.O.: project administration; N.I.: conceptualization and supervision; I.T.: data collection, data analysis; M.B.: writing – original draft; M.E.L.A.: review and editing.

## Conflicts of interest disclosure

The authors have no competing interests to declare that are relevant to the content of this article.

## Research registration unique identifying number (UIN)

1. Name of the registry: NA.
2. Unique Identifying number or registration ID: NA.
3. Hyperlink to your specific registration (must be publicly accessible and will be checked): NA.

## Guarantor

Mohammed Boutaybi.

## Provenance and peer review

Not commissioned, externally peer-reviewed.

## References

[1] Agha RA Franchi T Sohrabi C . for the SCARE Group. The SCARE 2020 guideline: Updating Consensus Surgical CAseREport (SCARE) guidelines. Int J Surg 2020;84:226–230. 33181358 10.1016/j.ijsu.2020.10.034

[2] Alegria JR Hermann J Holmes DR . Myocardial bridge. EUR Heart J 2005;26:1159–1168. 15764618 10.1093/eurheartj/ehi203

[3] Huang XH Wang SY Xu JP . Surgical outcome and clinical follow-up in patients with symptomatic myocardial bridging. Chin Med J (Engl) 2007;120:1563–1566. 17908470

[4] Kunamneni PB Rajdev S Krishnan P . Outcome of intracoronary stenting after failed maximal medical therapy in patients with symptomatic myocardial bridge. Catheter Cardiovasc Interv 2008;71:185–190. 18327835 10.1002/ccd.21358

[5] Corban MT Hung OY Eshtehardi P . Myocardial bypass: contemporary understanding of pathophysiology with implications for Am Coll Cardiol diagnostic and therapeutic strategies. J Am Coll Cardiol 2014;63:2346–2355. 24583304 10.1016/j.jacc.2014.01.049 PMC4065198

[6] Noble J Bourassa MG Petitclerc R . Myocardial bridging and milking effect of the left anterior descending coronary artery: normal variant or obstruction? Am J Cardiol 1976;37:993–999. 1274883 10.1016/0002-9149(76)90414-8

[7] Kursaklioglu H Barcin C Iyisoy A . Angiographic restenosis after myocardial bridge stenting. Jpn Heart J 2004;45:581–589. 15353869 10.1536/jhj.45.581

[8] Teragawa H Fujii Y Ueda T . Case of angina pectoris at rest and during effort due to coronary spasm and myocardial bridging. World J Cardiol 2015;7:367–372. 26131343 10.4330/wjc.v7.i6.367 PMC4478573

[9] Yu M Zhou L Chen T . Myocardia ischemia associated with a myocardial bridge with no significant atherosclerotic stenosis. BMC Cardiovasc Disord 2015;15:165. 26646509 10.1186/s12872-015-0158-2 PMC4673761

[10] Anderson KR Ho SY Anderson RH . Location and vascular supply of sinus node in human heart. Br Heart J 1979;41:28–32. 426954 10.1136/hrt.41.1.28 PMC514694

[11] García García C CurósAbadal A Serra Flores J . Duration of complete atrioventricular block complicating lower wall infarction treated with fibrinolysis. Rev Esp Cardiol 2005;58:20–26. 15680127

[12] Tans AC Lie KI Durrer D . Clinical setting and prognostic significance of high degree atrioventricular block in acute inferior myocardial infarction: a study of 144 patients. Am Heart J 1980;99:4–8. 7350750 10.1016/0002-8703(80)90308-7

[13] Giglioli C Margheri M Valente S . Timing, setting and incidence of cardiovascular complications in patients with acutemyocardial infarction submitted to primary percutaneous coronary intervention. Can J Cardiol 2006;22:1047–1052. 17036099 10.1016/s0828-282x(06)70320-8 PMC2568965

[14] Tandon A Simpson L Assar MD . Unusual origin of atrioventricular block type 1 with comments on Wenckebach’s contribution. Proc (Bayl University Med Cent) 2011;24:9–12. 10.1080/08998280.2011.11928674 PMC3012282 21307969

[15] Brignole M . Diagnosis and treatment of syncope. Heart 2007;93 ÿ 130–136. 17170354 10.1136/hrt.2005.080713 PMC1861366

[16] Bourassa MG Butnaru A Lespérance J . Symptomatic myocardial bridges: overview of ischemic mechanisms and current diagnostic and treatment strategies. J Am Coll Cardiol 2003;41:351–359. 12575960 10.1016/s0735-1097(02)02768-7

[17] Attaran S Moscarelli M Athanasiou T . Is coronary artery bypass grafting an acceptable alternative to myotomy for the treatment of myocardial bridging? Interact Cardiovasc Thorac Surg 2013;16:347–349. 23171516 10.1093/icvts/ivs459 PMC3568793

